# Validation of the self-reported domains of the Edmonton Frail Scale in patients 65 years of age and older

**DOI:** 10.1186/s12877-022-03623-1

**Published:** 2023-01-11

**Authors:** Luxey Sirisegaram, Oluwafemi P. Owodunni, April Ehrlich, Caroline Xu Qin, Dianne Bettick, Susan L. Gearhart

**Affiliations:** 1 The Johns Hopkins University Bloomberg School of Public Health, Baltimore, MD USA; 2 Schulich School of Medicine, University of Western Ontario, London, ON Canada; 3 Department of Surgery, The Johns Hopkins University School of Medicine, Baltimore, MD USA; 4 Department of Medicine, Johns Hopkins University School of Medicine, Baltimore, MD USA; 5 Department of Surgery, Johns Hopkins Bayview Medical Center, A Building, 4940 Eastern Avenue, Baltimore, MD 21286 USA

**Keywords:** Edmonton frail scale, Frailty, Preoperative

## Abstract

**Introduction:**

In the era of virtual care, self-reported tools are beneficial for preoperative assessments and facilitating postoperative planning. We have previously reported the use of the Edmonton Frailty Scale (EFS) as a valid preoperative assessment tool.

**Objective:**

We wished to validate the self-reported domains of the EFS (srEFS) by examining its association with loss of independence (LOI) and mortality.

**Methods:**

This is a post-hoc analysis of a single-institution observational study of patients 65 years of age or older undergoing multi-specialty surgical procedures and assessed with the EFS in the preoperative setting. Exploratory data analysis was used to determine the threshold for identifying frailty using the srEFS. Procedures were classified using the Operative Stress Score (OSS) scored 1 to 5 (lowest to highest). Hierarchical Condition Category (HCC) was utilized to risk-adjust. LOI was described as requiring more support at discharge and mortality was defined as death occurring up to 30 days following surgery. Receiver operating characteristic (ROC) curves were used to determine the ability of the srEFS to predict the outcomes of interest in relation to the EFS.

**Results:**

Five hundred thirty-five patients were included. Exploratory analysis confirmed best positive predictive value for srEFS was greater or equal to 5. Overall, 113 (21 percent) patients were considered high risk for frailty (HRF) and 179 (33 percent) patients had an OSS greater or equal to 5. LOI occurred in 7 percent (38 patients) and the mortality rate was 4 percent (21 patients). ROC analysis showed that the srEFS performed similar to the standard EFS with no difference in discriminatory thresholds for predicting LOI and mortality. Examination of the domains of the EFS not included in the srEFS demonstrated a lack of association between cognitive decline and the outcomes of interest. However, functional status assessed with either the Get up and Go (EFS only) or self-reported ADLs was independently associated with increased risk for LOI.

**Conclusion:**

This study shows that self-reported EFS may be an optional preoperative tool that can be used in the virtual setting to identify patients at HRF. Early identification of patients at risk for LOI and mortality provides an opportunity to implement targeted strategies to improve patient care.

## Introduction

In 2018 we implemented a Geriatric Surgery Pathway which aligns with the American College of Surgeon’s Geriatric Surgery Verification Program (ACS-GSV). As part of this pathway and following the recommendations of the ACS-GSV guidelines, we introduced a high-risk for frailty (HRF) assessment into our clinical workflow, which was performed on adults 65 years of age or older undergoing surgery [1]. Although the pervasive view of frailty is that it is a state of vulnerability to stressors, the lack of a universal definition of frailty provides a unique challenge in the measurement of it. Fried and colleagues developed the phenotypical model of frailty, which utilizes quantifiable physical ability of patients [2]. Frailty has also been defined as a risk index, where deficits accrued over time and are quantified using a Frailty Index [3]. The Edmonton Frail Scale (EFS) is a validated tool that was tested against the Geriatrician’s Clinical Impression of Frailty (GCIF) and it incorporates multidimensional clinical characteristics of patients, which are included in a comprehensive geriatric assessment [4]. We chose to utilize the Edmonton Frail Scale (EFS) in our clinical setting because of its comprehensive nature, as it incorporates a cognitive screen (clock-draw), a functional screen (Timed Get up and Go), as well as psychosocial dimensions of health as self-reported questions [4]. It is an 11-item scale, of which 9 items are self-reported [4, 5]. The EFS takes approximately only 3—5 min to complete, however it does require administration by a trained certified medical assistant during an in-person visit [4].

Recently, we demonstrated that pre-operative patients 65 years of age or older with an EFS score 6 or more were more likely to suffer loss of independence (LOI), an outcome often overlooked in this at-risk patient population [6]. However, LOI is often the outcome which matters most to older adults [7]. Moreover, we identified that the domains of the EFS most indicative of LOI included self-reported depression, weight loss, and limited mobility [6]. Pre-emptive identification of possible LOI is beneficial and utilizing tools for identification of patients in the preoperative setting in need of additional postoperative support is a proven mechanism to improve postoperative outcomes [8].

Classically, preoperative assessments are an in-person evaluation comprised of a standard assessment for patients undergoing a surgical procedure involving anesthesia [9]. Preoperative assessments have been associated with numerous positive benefits ranging from improved mortality, resource allocation, patient satisfaction and decreased surgical delay [10]. However in 2020, the novel coronavirus (COVID 19) pandemic introduced challenges into healthcare workflow. Routine perioperative care was particularly affected, and the pandemic made safe healthcare more difficult for our older frail patients. Furthermore, we noted a lack of virtual tools that could screen patients who were at HRF on the day of their visit. Given the recent changes to our perioperative workflow and evolving virtual care needs, we wish to evaluate the self-reported components of the EFS (srEFS) to capture patients at HRF and to aid in the prediction of postoperative outcomes, specifically regarding those at risk of LOI and mortality.

## Methods

### Study population

We performed a post-hoc analysis of an observational study that used the EFS as a preoperative frailty screen to assess the relationship between frailty and postoperative LOI [6]. This study included patients 65 years and older who were evaluated using our screening methodology in our surgical clinics prior to a surgical intervention. A certified medical assistant administered the EFS in the standard format in an in-person preoperative clinic, which was input into our institution's electronic health record system (EHR). All inpatient and outpatient procedures designated as elective were included, as were demographic and procedural characteristics**.** Inclusion criteria included patients 65 years or older who had a preoperative evaluation utilizing the EFS and who were undergoing an elective surgical procedure from June 2019 to June 2020. Exclusion criteria included patients who had undergone non-elective surgery or did not have an EFS completed. The Johns Hopkins University Institutional Review Board provided ethical approval, and all procedures and methodological guidelines were adhered to.

### Risk assessment indices

#### Edmonton frail scale

The EFS is a validated frailty measurement tool, with acceptable construct validity and reliability [4]. It is designed to evaluate aspects of the geriatric syndrome including cognitive decline, functional decline, poor nutrition and weight loss, social isolation/lack of support, and depression. In the present study, we focused on the self-reported domains of EFS (srEFS). Participants completed the entire EFS, however for the purpose of this study, the self-reported domains were evaluated independently. The self-reported domains include the following:

1. General health status was defined as the number of times a patient was admitted to the hospital in the previous year (scored 0–2 points), and the patient’s perception of their health (scored excellent/very good/good, fair, or poor).
2. Independence was defined as the inability to execute one or more ADLs, (scored 0–2 points).
3. Social support was defined as the ability to rely on someone who is willing and competent to meet their needs (scored always, sometimes, or never).
4. Polypharmacy was defined as using five or more prescription medications on a regular basis (scored yes, or no), as well as occasionally forgetting to take their prescription medications (scored yes, or no).
5. Nutrition was assessed as patients saying that their clothes felt loose (scored yes, or no)
6. Depression was defined as patients expressing feelings of "sadness" (scored yes, or no)
7. Incontinence was defined as a concern with not being able to control their bladder (scored yes, or no).

We performed summary measures for the desired list of cut-off values, including sensitivity, specificity, the proportion of cases correctly classified, positive predictive value, and likelihood ratios, to set the benchmark for srEFS used in characterizing frailty. These indicators were used to estimate the appropriate optimal decision threshold/cut-off values for srEFS. The stratum criteria for frailty on the srEFS was determined to be 5 or greater (Table 1)**.**

**Table 1:** Detailed Report on the Sensitivity and Specificity Thresholds for the Self-reported Edmonton Frailty Scale Cut-off

Threshold	Sensitivity	Specificity	Correctly Classified	Positive Likelihood Ratio	Negative Likelihood Ratio
(≥ 0)	100.0%	0.0%	24.1%	1	-
(≥ 1)	100.0%	11.1%	32.5%	1.12	0
(≥ 2)	100.0%	33.7%	49.7%	1.51	0
(≥ 3)	97.7%	63.6%	71.8%	2.68	0.04
(≥ 4)	96.1%	83.5%	86.5%	5.82	0.05
(≥ 5)	77.5%	96.8%	92.2%	24.21	0.23
(≥ 6)	45.0%	100.0%	86.7%	-	0.55
(≥ 7)	23.3%	100.0%	81.5%	-	0.77
(≥ 8)	11.6%	100.0%	78.7%	-	0.88
(≥ 9)	4.7%	100.0%	77.0%	-	0.95
(≥ 10)	2.3%	100.0%	76.5%	-	0.97
(> 10)	0.0%	100.0%	75.9%	-	1.00

### Operative stress score

The operative stress score (OSS) is a numerical scale ranging from 1 to 5 (lowest to highest) that corresponds to the level of physiologic stress expected to be experienced by the patient [11]. This score was generated and validated in a large patient population by Shinall et al. [11].

### Hierarchical condition category risk

The Centers for Medicare and Medicaid Services (CMS) employs Hierarchical Condition Category (HCC) Risk Scores as a risk-adjustment model to predict the cost of health-care services utilization for Medicare beneficiaries [4, 7]. The HCC risk score captures patients’ demographic characteristics such as age as sex, as well as diagnoses-based clinical measures to produce a risk-adjustment scoring system [12]. The International Classification of Diseases Ninth and Tenth Revision diagnosis codes are used to calculate HCC scores. CMS methodology is used to produce HCC scores, which are then entered into our EHR system. HCC scores were extracted for each patient and dichotomized into two main categories: HCC < 1 underpredicts healthcare utilization costs, and scores of 1 or above show a true or approximate value for the healthcare service. HCC is a tool to allow for risk adjustment for patient co-morbidities that appears to have no confounding effect with EFS results.

### Postoperative outcomes

Outcomes of interests included LOI at discharge and 30-day mortality. LOI was defined as a loss of functional independence reflective of changes in discharge location. A transfer of postoperative care to a facility of care higher than the preoperative status because of the need of additional supports (skilled nursing facility, or rehabilitation facility) was strictly defined as LOI. Mortality was defined as death within 30 days of having a surgical procedure performed.

### Statistical analysis

In this post-hoc analysis, all statistical analyses were carried out using STATA 15 (StataCorp, LLP, College Station, TX). We utilized threshold-based dichotomous classification studies to evaluate the revised numerical threshold for the cumulative scores for the srEFS domains. We ran non-graphical and graphical exploratory statistical analyses, starting with simpler descriptive analytic approaches and progressing to more refined parametric analytic methods, including projection methods. Receiver Operator Curves (ROC) were used to estimate the discriminatory thresholds of srEFS relative to LOI and mortality. Wilcoxon rank-sum tests were used to compare the medians for the baseline characteristics of patients, and Fisher’s and chi-squared exact tests were used to compare dichotomous and categorical data. The uncorrected and adjusted odds ratios (OR) for LOI were calculated using logistic regression models. Estimates for these models are reported as OR and corresponding 95% confidence intervals (CI). Bonferroni corrections tests were used to compare the areas under the curve for different instruments. A *p*-value 0.05 or greater was used as the significance criterion for all analyses.

## Results

The stratum criteria for frailty on the srEFS was determined to be 5 or greater based on its highest specificity (96.8%), correctly classified (92.2%), and with positive likelihood ratio of 24.2 (Table 1).

A total of 535 patients ≥ 65 years who completed the EFS assessment and underwent elective surgery were included. 113 patients (21.2%) were identified as HRF with an srEFS score of 5 or greater (Table 2). Compared to non-HRF patients, HRF patients were significantly older (71 vs. 75) and had a higher HCC score (163 vs. 70). There was no significant difference between sex, race, BMI, severity of surgery performed (based on OSS), or inpatient/outpatient status between non-HRF and HRF patients. Patients identified as HRF by the srEFS score of 5 or greater experienced higher rate of LOI than non-HRF patients (3.6% versus 20.4%).

**Table 2:** Patient Demographics and Clinical Characteristics Relative to the Self-reported Edmonton Frailty Scale

Characteristics	Total (*N* = 535)	srEFS < 5 Non-High Risk for Frailty (*n* = 422)	srEFS ≥ 5 High Risk for Frailty (HRF) (*n* = 113)	*P*-Value^λ^
Age, median (IQR)	72.0 (68.0, 77.0)	71.0 (68.0, 76.0)	75.0 (71.0, 79.0)	< 0.001
Sex, n (%)				
Male	320 (59.8)	252 (59.7%)	68 (60.2%)	1.00
Female	215 (40.2)	170 (40.3%)	45 (39.8%)	
Race, n (%)				
White	399 (74.6)	328 (77.7%)	71 (62.8%)	0.01
Black	106 (19.8)	72 (17.1%)	34 (30.1%)	
Other^a^	30 (5.6)	22 (5.2%)	8 (7.1%)	
BMI, n (%)				
< 25 kg/m2	164 (30.7)	123 (29.1%)	41 (36.3%)	0.17
≥ 25 kg/m2	371 (69.4)	299 (70.9%)	72 (63.7%)	
HCC, n (%)				
< 1	302 (56.5)	259 (61.4%)	43 (38.1%)	< 0.001
≥ 1	233 (43.6)	163 (38.6%)	70 (61.9%)	
OSS, n (%)				
< 3	356 (66.5)	280 (66.4%)	76 (67.3%)	0.91
≥ 3	179 (33.5)	142 (33.6%)	37 (32.7%)	
Patient Status, n (%)				
Outpatient	241 (45.1)	191 (45.3%)	50 (44.2%)	0.92
Inpatient	294 (54.9)	231 (54.7%)	63 (55.8%)	
LOS, median (IQR)	1.0 (0.0, 2.0)	1.0 (0.0, 1.0)	1.0 (0.0, 2.0)	0.57
Mortality, n (%)				
No	514 (96.1)	409 (96.9%)	105 (92.9%)	0.060
Yes	21 (3.9)	13 (3.1%)	8 (7.1%)	
LOI, n (%)				
No	497 (93.0)	407 (96.4%)	90 (79.6%)	< 0.001
Yes	38 (7.0)	15 (3.6%)	23 (20.4%)	

*srEFS* self-reported Edmonton frailty scale (≥ 5 considered frail), *OSS* Operative Stress Score, *IQR* Interquartile range, *LOS* length of hospital stay, *LOI* postoperative loss of independence

^a^Asian, Native Hawaiian/Pacific Islander, American Indian/Alaska Native, or unknown

^λ^*P*-value Chi Squared and exact (*n* < 10) tests for proportions and non-parametric Wilcoxon rank-sum tests for medians

**Fig. 1:**
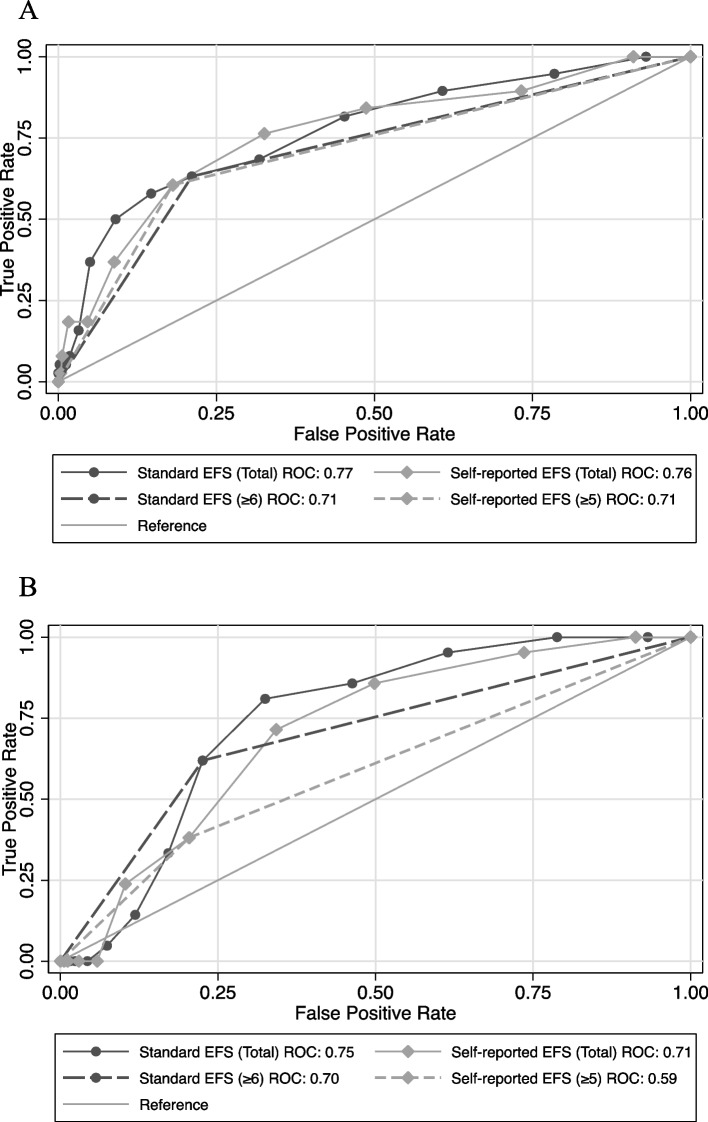
**A** The Discriminating Threshold on the Receiver Operating Characteristics Curve of Standard and Self-Reported Edmonton Frailty Scale Instruments for Loss of Independence at Discharge*. *Chi-squared and Bonferroni Corrections tests showed no differences for discriminatory thresholds across all ROCs (*p* > 0.05). **B** The Discriminating Threshold on the Receiver Operating Characteristics Curve of Standard and Self-Reported Edmonton Frailty Scale Instruments for Mortality*. **Chi-squared and Bonferroni Corrections tests showed no differences for discriminatory thresholds across all ROCs (*p* > 0.05)

The ability of the srEFS with the new HRF threshold 5 or greater to predict postoperative LOI and mortality as compared to the standard EFS is demonstrated in Figs. 1A and B. Figure 1A compares the area under the curve (AUC) for EFS and srEFS for the distinct ability of identifying LOI. The AUCs demonstrate that the standard EFS and the srEFS instruments have similar predictive ability for LOI (77% versus 76% respectively). A similar accuracy was observed for predicting mortality using the AUC for the standard EFS or srEFS, where the threshold score for frailty of EFS (6 or greater) and srEFS (less than or equal to 5) (75% versus 71%).

**Fig. 2:**
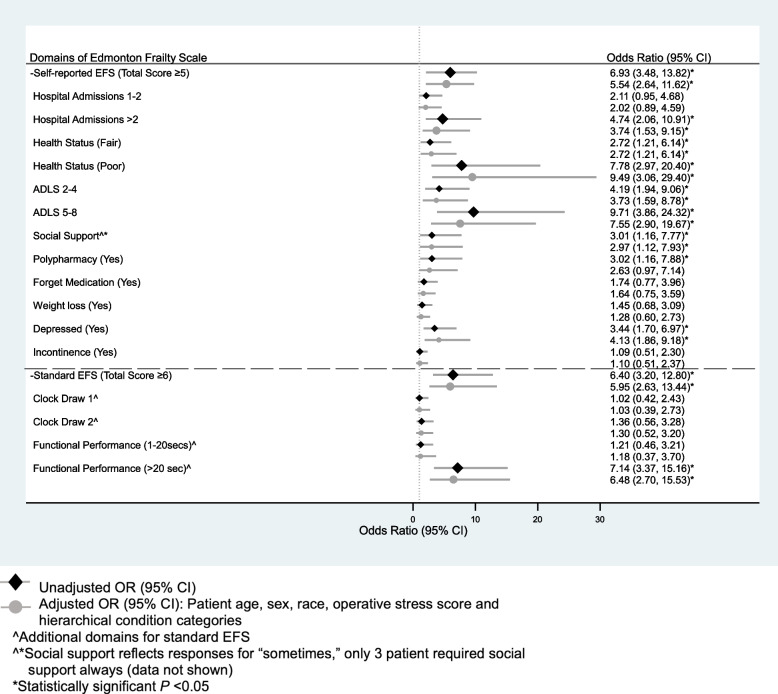
Univariate and Risk-Adjusted Analyses of Domains of the Standard and Self-Reported Edmonton Frailty Scales Associated with Loss of Independence at Discharge

Univariate and adjusted values of the both the standard EFS and the srEFS domains relative to loss of independence are shown in Fig. 2. The self-reported domains of two or more hospital admissions, health status rated as fair and poor, reduced ADLs to requiring assistance with 2–4 and 5–8 activities, lack of social support, and self-reported depression were associated with a significantly increased risk of LOI (Fig. 2). Interestingly, cognition assessed with the clock draw on the standard EFS was not associated with LOI.

## Discussion

Preoperative assessments for frailty have been shown to predict poor postoperative outcomes [13, 14]. Previously, Owodunni et al. examined the use of the standard EFS and demonstrated that patients classified as frail were at increased risk for LOI and mortality [6]. With the recent changes in healthcare delivery and clinical workflow, our study was aimed at investigating the self-reported domains of the EFS to determine if it would have a similar ability to predict postoperative LOI and mortality as the standard EFS. Our results demonstrate that under extenuating circumstances, where in-person visits are not possible, healthcare providers can utilize the srEFS, with a threshold score of 5 or more indicating HRF, to aid in the prediction of postoperative LOI with similar predictive ability as the previously validated standard EFS.

Additionally, it is important to note that only increased time on the Get Up and Go measured on the standard EFS was independently associated with LOI, while cognitive impairment measured on the Clock Draw was not. This suggests that in ideal practice conditions, the standard EFS would be the preferred tool to capture physical frailty associated with LOI**.** The clock draw is a screening test used to help diagnose cognitive impairment given its ability to test numerous cognitive domains including memory, visuospatial, concentration, motor function, perception, numerical knowledge etc [15, 16]. It has been demonstrated to be > 85% sensitive for cognitive impairment when used in the MMSE [17]. However, there have been studies that have shown the number of cognitive impairment cases amongst frail patients was not clearly reported [18–20]. Similarly, 22% of Alzheimer dementia patients have lacked physical indicators of frailty [21]. The International Association of Gerontology and Geriatrics/International Academy on Nutrition and Aging have proposed the concept of cognitive frailty to address this patient population [22, 23]. In the preoperative setting, studies have found mixed utility in the use of clock draw to identify preoperative patients at risk of developing delirium [24, 25]. Other tools that capture frailty, such as the Reported Edmonton Frail Scale (REFS), also include the clock draw as a component of testing, however a key difference is that the REFS is used in the acute inpatient setting, which may indicate that cognitive frailty may have a larger role in inpatient acute medicine in comparison to the preoperative elective surgery population [4]. In our study, the elimination of the clock draw was not associated with decreasing the predictive ability for LOI. This suggests that physical frailty, which describes a high-risk disposition with increased susceptibility to surgical stressors and low reserve, may be more important for the outcome of LOI [26]. It is important to note that we did not study the outcome of post-operative delirium, which is a vital aspect of surgical care, and thus in ideal practice conditions it may be important to use the standard EFS or REFS if evaluating risk for delirium.

As Nidadavolu et al. describe, there are a variety of preoperative frailty assessment tools that have been validated, however there is no gold standard for preoperative frailty assessments [27]. They also found that the majority of preoperative frailty assessments currently have an in-person component [27]. Although there are numerous self-reported frailty assessments that have been described in the literature, such as the REFS, which focuses on the acute inpatient setting, our goal was to explore tools that could be easily administered in the preoperative setting for elective surgical procedures [28]. Moreover, the REFS includes the clock-draw component of the EFS, which requires either an in-person assessment, or access to a camera and knowledge of how to use secure internet-based video software, which may be challenging for patients [28].

Under the current Age Friendly Health System framework put forth by the John A. Hartford Foundation, there has been a shift in provider focus to “what matters most” to the patient when planning a surgical intervention [29, 30]. The potential for LOI is now considered as deterrent by providers that may prolong quantity over quality of life, particularly in the area of interventions for cancer care [31]. Therefore, identifying risk factors for postoperative LOI have been a focus of recent investigations. Blankenship et al. recently delineated risk factors for postoperative LOI following pelvic organ prolapse in the hopes of improving shared decision making prior to surgery. They found that there was a higher risk of loss of functional independence for patients who were 80 years old or greater, had higher ASA scores and increased LOS [32]. Bonicoli examined outcomes at 2 years following hemiarthroplasty vs. osteosynthesis in patients 80 years or older who suffered hip fracture [33]. Their study demonstrated an increase in mortality risk following hemiarthroplasty but no difference in functional performance measures by the activities of daily living [33]. Finally, Bal et al. examined results of emergent inguinal hernia repair given that current recommendations have encouraged watchful waiting in elderly patients with an inguinal hernia [34]. His study reviewed results following emergent inguinal hernia repair in patients 70 years and older using the National Surgery Quality Improvement Program (NSQIP) database and demonstrated that elective surgery in these patients were associated with improved outcomes, including a lower likelihood for LOI or mortality [34].

Our results demonstrate the novel utilization of the srEFS to identify HRF patients, which can be feasibly performed via telephone interviews or virtual methods such as online patient health portals [35]. The shift in healthcare workflow has led some investigators to create virtual clinics for preoperative assessments. Joughin et al. recently described implementation of their virtual geriatric perioperative medicine clinic [36]. Comprehensive geriatric assessments were converted to questionnaires which could be utilized during a virtual visit. Patient survey results following implementation demonstrated optimism over the virtual methodology, particularly in the area of shared decision making [36]. Hands et al. found that alternative management plans that occurred because of telemedicine proved to be advantageous for both patient and consultants [37]. Similarly, our study highlights that srEFS may be used across a variety of surgical procedures given the diversity of surgical procedures studied.

There are several limitations to this study. The self-reported aspects were taken from our standard EFS evaluations that were already implemented in our preoperative clinic. They were provided as a paper questionnaire to the patient to answer in -person or the patient may have been asked the self-reported questions by a clinic provider. This data was then taken and entered into our institution’s EHR. The use of this data allowed for the timely evaluation using the srEFS, however additional studies examining the use of the srEFS in a virtual setting are warranted. In addition, patients who had an EFS performed up to 6 months prior to surgery were included and their frailty status may have changed in the time prior to surgery. We also included outpatient procedures which are not commonly associated with LOI, however we felt that these procedures are often performed in older patients at risk for LOI. Our data demonstrated that 20% of patients experiencing LOI had an outpatient procedure performed. This study is a single centre study, and confirmation of our results at other institutions is needed. Another limitation is that the EFS was completed by all patients who had undergone surgery including those who had cognitive impairment or other disabilities, which may have impacted their performance on their assessment. However, the EFS was chosen because it integrates many psychosocial aspects into the scoring system and thus is not heavily weighted by a patient’s disability or cognitive impairment. We included patients who required assistance with the clock-draw, or who could not perform an aspect of the test secondary to a disability to enhance its generalizability to all older adults. Another limitation is the heterogeneity of the population of this study, given that both inpatient and outpatients were included. However, we attempted to control for this heterogeneity by including OSS and HCC into our analysis. Finally, this study used the ACS NSQIP® registry database, which records solely 30 day mortality post-surgery [38]. Moreover, it lacks specific information regarding comorbidities, their impact on functioning, surgical outcomes or reason for surgery [38]. Additionally, ACS- NSQIP does not differentiate between unique events that are specific to certain operations, which may have been useful in this study to analyze which specific events had an impact on mortality and LOI. However, ACS-NSQIP is currently creating a procedure-specific form that is aimed at a specific procedure and collecting results and variables [39].

Future directions of this study include exploring the associations between the srEFS being done virtually or using telehealth, and its impacts pre and postoperative planning. We anticipate targeting domains of the srEFS that have been shown to increase risk for LOI. Furthermore, we hope to also apply the srEFS in acute care or emergency care, where it can be completed in emergent/urgent way and allow for early identification of patients at risk for LOI.

## Data Availability

Availability of Data and Material (ADM)—The datasets generated and analyzed during the current study not publicly available due to privacy, however they are available from Dr. Susan Gearhart on a reasonable request.
